# Acceptability of automatic referrals to supportive and palliative care by patients living with advanced lung cancer: qualitative interviews and a co-design process

**DOI:** 10.1186/s40900-024-00568-0

**Published:** 2024-04-02

**Authors:** Sadia Ahmed, Jessica Simon, Patricia Biondo, Vanessa Slobogian, Lisa Shirt, Seema King, Alessandra Paolucci, Aliyah Pabani, Desiree Hao, Emi Bossio, Ralph Cross, Tim Monds, Jane Nieuwenhuis, Aynharan Sinnarajah

**Affiliations:** 1 Alberta SPOR SUPPORT Unit, Patient Engagement Team, 3280 Hospital Dr NW, Calgary, AB T2N 4Z6 Canada; 2 Department of Community Health Sciences, University of Calgary, 3280 Hospital Dr NW, Calgary, AB T2N 4Z6 Canada; 3 Division of Palliative Medicine, Department of Oncology, Cumming School of Medicine University of Calgary, Calgary, AB Canada; 4 Department of Oncology, Cumming School of Medicine University of Calgary, Calgary, AB Canada; 5 Palliative and End of Life Care, Alberta Health Services, Calgary Zone, Calgary, Canada; 6 Patient and Family Advisor, Alberta Health Services, Calgary, AB Canada; 7 Division of Palliative Medicine, Department of Medicine, Queen’s University, Kingston, ON Canada

**Keywords:** Palliative care, Patient engagement, Co-design, Early referral process, Perceived acceptability, Advanced lung cancer

## Abstract

**Purpose:**

Timely access to supportive and palliative care (PC) remains a challenge. A proposed solution is to trigger an automatic referral process to PC by pre-determined clinical criteria. This study sought to co-design with patients and providers an automatic PC referral process for patients newly diagnosed with stage IV lung cancer.

**Methods:**

In *Step 1* of this work, nine one on one phone interviews were conducted with advanced lung cancer patients on their perspectives on the acceptability of phone contact by a specialist PC provider triggered by an automatic referral process. Interviews were thematically analysed. *Step 2*: Patient advisors, healthcare providers (oncologists, nurses from oncology and PC, clinical social worker, psychologist), and researchers were invited to join a working group to provide input on the development and implementation of the automatic referral process. The group met biweekly (virtually) over the course of six months.

**Results:**

From interviews, the concept of an automatic referral process was perceived to be acceptable and beneficial for patients. Participants emphasized the need for timely support, access to peer and community resources. Using these findings, the co-design working group identified eligibility criteria for identifying newly diagnosed stage IV lung cancer patients using the cancer centre electronic health record, co-developed a telephone script for specialist PC providers, handouts on supportive care, and interview and survey guides for evaluating the implemented automatic process.

**Conclusion:**

A co-design process ensures stakeholders are involved in program development and implementation from the very beginning, to make outputs relevant and acceptable for stage IV lung cancer patients.

**Supplementary Information:**

The online version contains supplementary material available at 10.1186/s40900-024-00568-0.

## Introduction

People living with advanced lung cancer often experience high symptom burden and emotional distress. However timely access to supportive and palliative care services that provide a comprehensive and person-centred approach to addressing their physical, psychological, social and spiritual suffering, remains a challenge [1, 2].

Multiple randomized controlled trials that have compared early palliative care concurrent with cancer care to usual care alone have provided evidence for early integration of PC with cancer care [3]. Using specialist palliative care (PC) services (consults by clinicians with PC training), early and concurrently with cancer modifying therapies (e.g. within 8 weeks of diagnosis of advanced cancer), has been shown to improve cancer patients’ quality of life, [4] caregiver outcomes, [3, 5] and health system sustainability [6], although with some heterogeneity in the settings and the results [7]. However, late referrals remain all too common, for a multitude of reasons [8, 9]. Oncology clinicians have many competing priorities to address in brief appointments, and they have limited time and physical space to evaluate and address their patients’ supportive and palliative care needs or have conversations with them about the benefits of using specialist services [10]. Additional barriers include stigma around the term ‘palliative care’ [2, 5, 11, 12] and inconsistencies in clinician referrals due to varied education, experience, interest, and understanding of PC [13, 14]. There are a range of prognostic trajectories in lung cancer, with different treatment options [15, 16]. Randomized trials of early PC relied on oncologists to approve case selection and introduce the study to patients, potentially biasing those who were approached [3]. In our prior intervention study aimed at integrating PC with metastatic colorectal cancer management (www.pacesproject.ca), we were able to cue oncologists and increase the proportion of patients receiving timely, early referrals for supportive and palliative care by 17%; however, continued barriers (e.g. competing priorities) remained a challenge for sustaining earlier referrals [10, 17]. Consequently, our oncology clinician partners asked, “Why can’t the palliative care team directly offer patients early supportive and palliative care referrals?”.

Therefore, we sought to build an automatic referral process for patients newly diagnosed with advanced (stage IV) non-small cell lung cancer that did not depend on busy oncologists to screen or even approve the offer of a palliative care consultation. We conceived that such a process could use specific clinical data in patients’ charts (e.g. referral form, progress notes) and/or electronic medical record (EMR) to identify patients eligible for an early palliative care consult. A PC provider could then contact the identified patient directly and offer them a consult. This could occur regardless of whether the oncology staff offered or even mentioned a palliative care referral. Before developing or implementing such a process, we needed to know how acceptable a direct phone call from a palliative care clinician would be to patients. Patient-oriented research engages patients and caregivers as partners in the research process [18]. Within palliative care research, only three articles have been published that describe how they have engaged patients as partners [19]. This paper reports a patient-oriented co-design process which included working with patient and family advisors, clinicians, and researchers to build the automatic referral process.

## Methods

This patient-oriented, qualitative project consisted of two steps: 1) a qualitative, thematic analysis [20] of the perspectives of patients living with advanced lung cancer on their cancer experiences and the idea of automatic PC referrals and 2) a co-design process [21] that engaged patient and family advisors, healthcare providers (oncologists, nurses from oncology and PC, clinical social worker, psychologist), and researchers in the development of an automatic referral process to supportive and palliative care. The University of Calgary Health Research Ethics Board of Alberta Cancer Committee approved the two steps of this project (HREBA.CC-20–0222 and HREBA.CC-21–0026).

## Theoretical framework

The Canadian Institutes of Health Research (CIHR) Strategy for Patient-Oriented Research (SPOR) Framework [18] guided the development and conduct of this project. The guiding principles of the framework are inclusiveness, support, mutual respect, and co-build. Inclusiveness aims to integrate a diversity of patient perspectives and research being reflective of the contributions of research. Support refers to creating safe environments that promote honest interactions, cultural competence, training, and education. Mutual respect means researchers, patients, and clinicians valuing each other’s expertise and experiential knowledge. Co-build refers to patients, researchers, and clinicians working together from beginning to end.

Additionally, Sekhon’s Theoretical Framework of Acceptability (TFA) [22] for healthcare interventions served as another framework for understanding the acceptability of an automatic referral process. The seven domains of the TFA are affective attitude, burden, ethicality, intervention coherence, opportunity costs, perceived effectiveness, and self-efficacy [22].These seven domains were used to guide qualitative question development and analysis in phase 1 of the project and the evaluative questionnaire for acceptability of the process developed in step 2.

## Context

Patients newly diagnosed with lung cancer in the study region are served by publicly funded [19] medical and radiation oncology outpatient clinics at a tertiary cancer centre, which serves 1.4 million people in a zone (Calgary, Alberta, Canada) including a metropolitan city, surrounding towns, rural areas and First Nations territories. A Lung Tumour Group Coordinator (nurse) triages new referrals to six lung-focused oncologists, and each oncologist clinic has one oncology nurse. In the cancer centre, Supportive Oncology services with psychosocial counsellors, social workers, and rehabilitation therapists are available upon referral or self-referral. Specialist palliative care services provide an added layer of support for patients (and their families) in addressing physical, psychological, social, and spiritual concerns. Within our region, this includes discussing with the patient (and family members) about their cancer journey, their wishes for their treatment, addressing and helping them with any symptoms they may be facing (e.g. pain management), and providing them with resources in the community or within the hospital setting to address their needs. Specialist palliative care is available across the continuum of care, with palliative physician and nurse specialist consults available in the cancer centre, hospitals, home and community settings; one tertiary palliative care inpatient unit and seven residential hospices. Homecare is available by referral or self-referral as dedicated, multidisciplinary palliative home care service in urban areas or as an “integrated” (general adult home care) service in rural areas.

## Step 1: Qualitative interviews with advanced lung cancer patients to understand their cancer experience and potential acceptability of an automatic referral process

We conducted a qualitative study through semi-structured, one-on-one interviews with patients living with advanced lung cancer (stage IV non-small cell lung cancer) to elicit and understand their perspectives on the idea of an automatic referral process for palliative care. The interview guide was developed in collaboration with two patient and family advisors and palliative care specialist clinician-researchers on the team. Sekhon’s Theoretical Framework of Acceptability (TFA) for healthcare interventions [22] was used to guide the development of interview questions and the analysis of participants’ responses. The interview guide consisted of questions about how they might perceive an automatic referral to early palliative care when they were newly diagnosed with advanced cancer, and what benefits and challenges this would bring to patients.

### Recruitment

Adult patients living with advanced lung cancer were eligible to be interviewed as participants. Patients deemed inappropriate by clinic staff to be approached for a study for any reason (e.g., in crisis) were excluded, as were patients who had already been seen by a supportive and/or palliative care provider. Potential participants were recruited through convenience sampling, through the lung tumour group coordinator at the cancer centre, posters posted near outpatient clinics, and patient support groups. We recruited participants until the start of step 2- our co-design process.

### Data collection & analysis

The qualitative researcher (first author identifies as a woman, MSc trained in qualitative and patient-oriented research) conducted interviews with consenting participants between September 2020 and March 2021. The researcher conducting the interviews did not have any prior relationships established with any of the study participants. Prior to starting the interviews, the researcher explained the project goals and reasons for doing the research. The qualitative researcher has prior early palliative care research experience and works in patient-engaged research, routinely working with patients as research partners.

All interviews were conducted via phone and/or video conferencing, audio-recorded, transcribed, and anonymized. Three researchers, all with qualitative research expertise and two with palliative care clinical experience conducted a thematic analysis of the transcripts utilizing deductive and inductive coding strategies. The 6-step Braun & Clarke [20] thematic analysis consisted of familiarizing ourselves with the data, and identifying initial codes. The seven dimensions of the TFA were used as a guide for the deductive coding scheme. Themes were identified, reviewed, defined and named. Member-checking occurred with presentation of findings to co-design working group.

## Step 2: Co-design

Research co-design is defined as the meaningful involvement of research users during the study planning phase of a research project [21]. We used a co-design method gathering patients, family members, healthcare providers, and researchers to develop a process of automatic palliative care referral to then pilot and evaluate in our cancer centre. This method’s strength lies in its ability to create a safe, inclusive, and respectful process through which all viewpoints and ideas can be considered. The aim of this approach was to establish a collaborative team environment for the development this process and to ensure the end product developed was meaningful and acceptable to the end-users. The objectives of the group were to co-design the automatic referral process, with the communication and operational pieces required for the automatic phone call program (e.g. criteria for referral, timing of the referral, supporting materials such as scripts for healthcare providers), as well as the instruments (i.e., interview guide and survey for patients) to evaluate the acceptability of the program. The subsequent implementation of the program and evaluation results will be published elsewhere and is outside the scope of this paper.

A terms of reference document was developed and shared with all co-design members to outline the purpose of the group, timelines, communication processes, and roles of the group. Additionally, the terms of reference document highlighted the core principles in building research partnerships to guide meeting conduct as stated by the Canadian Institutes of Health Research (CIHR) strategy for patient-oriented research framework: 1) mutual respect for different ways of knowing and interacting; 2) equitable participation and rights; 3) reciprocity and a shared commitment to producing relevant research results; and 4) personal integrity [23].

### Recruitment

We invited healthcare providers caring for lung cancer patients to join our co-design working group. Patient and family advisors from previous palliative and cancer care projects were also invited, including some of the patients interviewed in step 1 of this project. Our co-design group included 10 healthcare providers (oncologists, oncology nurses, specialist palliative care nurses and physicians, a clinical social worker and psychologist), 5 researchers, and 6 patient and family advisors (21 women, 5 men). The health care professionals who joined the working group were all affiliated with the cancer centre and had greater than five years of professional experience. Two of the six patient and family advisors were family members of deceased advanced cancer patients, while four were advanced lung cancer patients. They were orientated as patient/family advisors by Alberta Health Services volunteer services.

### Meeting logistics

The co-design group met biweekly via Zoom over the course of six months to collaboratively design the operational processes and communication pieces for automatic PC referral for newly-diagnosed stage IV lung cancer patients. Approximately fifteen co-design members attended each meeting.

## Results

### Results from step 1 – qualitative interviews

Nine patients living with advanced lung cancer were recruited and interviewed (3 women, 6 men). We recruited until the start of step 2: co-design phase of the project. Eight of nine patients had a stage IV diagnosis, and one patient had a stage III diagnosis but was deemed appropriate to be interviewed as they were at an advanced stage. Their ages ranged from 48 to 78 years. All participants identified as Caucasian/white. Interviews ranged from 21 to 65 min.

### Themes

Participants described their experiences with their cancer, any supports they had been accessing or desired since their diagnosis, and provided recommendations on how supportive and palliative care should be described to patients. As a result, three major themes were identified from our thematic analysis: *Emotional reactions to diagnosis*, *Current supports*, and *Perceptions of an automatic referral for palliative care*. Table 1 provides an overview of the themes, description, and supporting quotes identified.

**Table 1:** Qualitative Interviews: Themes Identified

Theme	Description	Supporting Quotes
**Emotional reactions to diagnosis**	Some Participants highlighted uncertainty and stress leading up to their diagnosis and following the investigative testing	“I was quite devastated actually. And especially when the doctor tells me to, “Get your affairs in order” and Stage 4 lung cancer is not something that is survivable; it is terminal. And yeah, so it’s been a bit of a challenge that’s for sure” (patient 1)
**Current Supports**	Early supports mentioned by participants as important were information on resources and services that are available to patients, symptom management, social/emotional support, and information on what to expect in cancer care and treatment Some patients were already seeking out certain supports such as online support groups, exercise programs, and smoking cessation programs. One patient mentioned the need for transportation to the cancer center, and another patient mentioned that home support, such as with meal preparation, would be helpful for patients. Participants mentioned that having access to social support, whether informally through their friends and families or though formal support groups, was vital for patients	“Now, I had a whole bunch of questions on things like how could I – things like sleeping, you know. What are the tricks that could get me to sleep? What about my appetite? And what type of resources would there be to kind of have a more positive outlook? What kind of support out there? Is there anybody out there like me that I could talk to? All those kinds of things, I could have used those right away.”(patient 4) “I don't know what I would have done without her [clinical social worker] contact. It was just so helpful…she was telling [me] what days my testing was back, when I could expect to hear, things that are really, really critical in those initial days. I don't know what I would have done without that because, as I was saying, nobody at [the cancer centre] was calling me back.” (patient 2)
**Perceptions of an automatic referral process for palliative care**	When asked about their thoughts on the automatic referral process, patients were supportive of the idea, and highlighted that many early needs of patients could be met with such a program. For instance, a phone call following a patient’s diagnosis could help in guiding future decision-making Patients also highlighted important components of the phone call necessary to communicate with patients. For instance, one patient described the need to frame and explain the call as part of the cancer care plan One challenge raised by some interviewees was that some patients may feel overwhelmed and may not feel they need a palliative care consultation right after their diagnosis:	“Having the opportunity to talk to a doctor or a nurse one-on-one, right, you having supportive care is very important and it means a lot to people… Now chemo, I didn’t know exactly what it was because this was my second tumor, and it was very painful both of them… she called me one day and she said, “It’s worth a talk” so that gave me a second thought right there ‘well maybe there is something good’ because I didn’t look up chemo until I was right in it.”(patient 8) “I would want to know, first of all, how did you get my name. So, I think I'd want somebody to say, so as part of the process, if your oncologist didn’t have the chance to tell you, we are a follow-up call to check in with you. We’re here to provide supportive connections, answers, people that you can access.” (patient 5) “I think the only challenge would be that a patient might not be as receptive to it, or they might not be in a place yet where they’re receptive. But even if the outreach was made and then there was information given that if the patient felt ready. I also think like a gentle follow up would actually – for patients who didn’t sort of initially bite, I think that would be really important as well, because there’s so much going on when you receive that kind of shocking diagnosis. It really is like a PTSD, frankly." (patient 2) “Maybe don’t even use it [palliative]. I don't know. If you have to, maybe not in the first phone call. Maybe you could use it in a subsequent phone call, once you’ve explained what it actually is.” (patient 3)

#### Theme: Emotional reactions to diagnosis

Under this theme, participants described the journey of receiving their diagnosis, and with the waiting period to hear news from the cancer centre. Some participants highlighted uncertainty and stress leading up to their diagnosis and following the investigative testing. For instance, one patient spoke of not receiving any news from the cancer centre for three weeks about their investigative testing but were relieved when a clinical social worker from the cancer centre reached out:

“*I don't know what I would have done without her contact. It was just so helpful…she was telling [me] what days my testing was back, when I could expect to hear, things that are really, really critical in those initial days. I don't know what I would have done without that because, as I was saying, nobody at [cancer centre] was calling me back*.” (patient 2).

For some participants, they mentioned the shock of receiving their diagnosis, especially for those patients who mentioned they were non-smokers.

#### Theme: Current supports

Participants mentioned early supports that are necessary for cancer patients, such as information on what current resources are available, support for symptom management, social/emotional support, and information on what to expect for their cancer care.

One patient spoke about the different questions they had and supports they needed:

“*Now, I had a whole bunch of questions on things like how could I – things like sleeping, you know. What are the tricks that could get me to sleep? What about my appetite? And what type of resources would there be to kind of have a more positive outlook? What kind of support out there? Is there anybody out there like me that I could talk to? All those kinds of things, I could have used those right away.* “(patient 4).

Some patients were already seeking out certain supports such as online support groups, exercise programs, and smoking cessation program. One patient mentioned needing transportation to the cancer center, and another patient described support in the home such as with meals would be helpful for patients. Participants reported having access to social support as vital for patients, whether informally through their friends and families or though formal support groups.

#### Theme: Perceptions of an automatic referral process for PC

When asked about their thoughts on the automatic referral process, patients were supportive of the idea, and highlighted that many early needs of patients could be met with such a program. For instance, a phone call following a patient’s diagnosis could help in guiding future decision-making for treatments. Participants highlighted the need for early support which was also emphasized through the previous themes. Important components of the phone call included the need to frame the call as part of cancer care, and to ensure patients knew the healthcare provider calling them was a member of their cancer care team.

A challenge perceived by some participants was the overwhelming experience of receiving an advanced cancer diagnosis, which may deter certain patients from accepting PC support, at the time of diagnosis, if the person views this as end of life care. For instance, one patient mentioned:

“*I think the only challenge would be that a patient might not be as receptive to it, or they might not be in a place yet where they’re receptive. But even if the outreach was made and then there was information given that if the patient felt ready. I also think like a gentle follow up would actually – for patients who didn’t sort of initially bite, I think that would be really important as well, because there’s so much going on when you receive that kind of shocking diagnosis. It really is like a PTSD, frankly*." (patient 2).

Current gaps within cancer care were described by participants in both the theme of *emotional reactions to diagnosis* and *current supports*. In describing the theme *automatic referral process for PC,* patients emphasized how such a process could be helpful: in providing early supports and comfort for patients receiving an advanced cancer diagnosis.

### Results from step 2 – co-design process

Findings from step 1 of this work informed the discussions and guided many of the decisions of the co-design working group. The co-design working group discussion topics and products are outlined in Table 2. The outputs of the co-design process have been uploaded on our project website [24]. During the initial meetings, patient and family advisors spoke of their own experiences of navigating the healthcare system once they had done the initial investigative tests, awaiting their diagnosis, and following their diagnosis. This informed the patient journey and a map of current services. Then, the co-design working group developed the communication and operational pieces for the automatic phone call program, as well as the instruments (i.e., interview guide and survey) to evaluate the program. The co-design group acknowledged and discussed the very varied prognoses within NSCLC in the co-design process. They decided that all patients could benefit from the offer of a consult, from those with cancer mutations for which there are targeted therapies and longer survival, to those with a poor prognosis, and for patients who would have further cancer centre visits and those who would not be seen there again.

**Table 2:** Co-design Working Group discussion topics and timeline

Content of Meetings	Month 1	Month 2	Month 3	Month 4	Month 5	Month 6
Mapping the patient journey	x					
What are the existing early supports? Patient needs?	x					
Naming: What do we call the support?		x				
How are eligible patients identified? How do we send the list of eligible patients to the palliative care provider?		x				
Timing of the call		x				
Phone calls in other languages for non-English speaking individuals?			x			
Developing a poster to notify patients about the call			x			
Developing scripts for oncology clinicians and palliative care providers			x			
Posters/ handouts to share about the program and community resources			x	x	x	
Process diagram and validation with group					x	
Evaluation of the research call – survey and interview guide						x

#### Naming the service

Various perspectives on the meaning associated with the term ‘palliative care’ were shared when discussing what to name this service. Similar to the views expressed in the interviews, some patient advisors on our team discussed that hearing the term ‘palliative care’ can be quite shocking for patients, especially when they hear it for the first time. Some patient advisors mentioned the stigma associated with PC may disengage patients during the phone call and may also deter them from accepting a consult. Other co-design members emphasized the importance of normalizing and de-stigmatizing the term PC through education. The term ‘supportive care’ was also discussed as a possible alternative name. Our cancer centre provides Supportive Care services, which are comprised of psychological counselling, psychiatry, rehabilitation services, social work and nutrition services. The regional PC services are provided as an added layer of support tending to physical, social, psychological and spiritual/existential needs and symptoms by healthcare providers with additional PC training. While there can be overlap between the two services, some co-design members expressed concern that confusion may arise among healthcare providers at the cancer centre, if the term ‘supportive care’ is used alone to describe both services. Co-design members respected the different views on the term for the program, therefore, members advised on using “Supportive and Palliative Care” to describe the automatic referral program.

#### Timing of the call

Co-design group members discussed and agreed that all patient groups needed to be called and offered a consult for PC supports, as soon as possible (within two weeks) after the first oncology visit. The co-design group agreed that the first oncology visit would likely be overwhelming for patients, as they are receiving information about their diagnosis and possible treatment plan. Therefore, a call from the specialist PC nurse after the first oncology visit would help to attend to any anxiety and provide reassurance of supports available for patients.

#### Supporting materials

Co-design members discussed a process for the oncology team to inform patients that they may be receiving a phone call from a specialist PC nurse. Members mentioned the need for a poster or handout alongside a conversation with the oncologist or oncology nurse during the first oncology visit to signify the importance of the call or aid in recall, given the often overwhelming emotional nature and amount of content at an initial cancer visit. The members co-developed a patient handout for distribution at the first oncology visit. However, members discussed that it would be acceptable to deliver the phone call even without notifying patients (if oncology healthcare providers forget or miss the opportunity to mention the consult and give the handout).

Members also discussed the importance of sharing resources about early supports available with patients. Patient and family advisors in the group expressed how peer support and community groups, including lung tumor-specific information groups, helped them in coping with their illness. The co-design working group co-developed a “message of hope” and a handout on support groups, websites, and information on exercise and mental health supports that would be most beneficial for newly diagnosed patients. Subsequently, in collaboration with the patient and family advisors and psychologists in the co-design group, as well as the Patient Education team at the cancer centre, we adapted the resources for online dissemination on the provincial website for cancer care [25].

#### Scripts

A script for oncology clinicians to notify patients about the automatic referral and for the specialist PC nurse (when making the initial phone call) was co-developed through multiple revisions. This script outlined the purpose of the call, personalizing the call to the patient (referring to their chart/immediate needs), defining “Supportive and Palliative care”, describing the consult, and informing the patient that the resources can be mailed/emailed to them for further support.

#### Evaluation of acceptability

The co-design team reviewed and discussed the draft survey and interview guide (Appendix 1) for evaluating the acceptability of the automatic phone call. It was decided that the survey and interview guide would be administered by a research team member and not the PC nurses providing the service to avoid potential participant response biases. Revisions were made to improve the length of the survey and wording of the questions.

After compiling all the communication pieces and logistical details of the automatic phone call process, a process diagram was presented to the co-design group for feedback, refinement, and final approval [24]. The final process diagram includes considerations for patients unable to speak with the specialist PC nurse, for example due to cognitive impairment or language barriers [24]. To address these barriers, the specialist PC would ask to speak to the caregiver/family member and/or language interpretation would be provided for those with language barriers (Appendix 2).

## Discussion

Informed by the qualitative interview themes, the co-design process successfully yielded all the deliverables to build a process to offer a specialist PC consultation to every single eligible patient (regardless of oncology opinion or referral) following a first visit to an oncologist with a new diagnosis of stage IV lung cancer. The group also co-designed the evaluation methods to be used to test the acceptability of this automatic referral.

Research co-design, patient engagement, and stakeholder engagement encompass similar strategies and often are used interchangeably in the literature. A rapid review of reviews identified various health research co-design approaches, and noted there to be a lack of a single, consistent conceptualization of co-design [21]. The authors identified many activities involved in research co-design, and the limitations of poorly defined engagement strategies or the ways in which they involved their stakeholder groups, as well as the outcomes of the engagement [21]. Without a standard framework for co-design, our project followed the CIHR strategy for patient-oriented research framework and ethical guidance for research partnerships to engage our co-design working group members [18, 23]. We partnered with patient and family advisors as well as healthcare providers throughout the planning phase of the automatic referral process. Co-design members were involved in the entire intervention planning process, including the evaluation of the program and the development of knowledge translation outputs (such as digital stories, presentations, and manuscripts), [24] which we believe was essential to creating an acceptable method of automatic palliative contact with people living with lung cancer.

In a Delphi study, international PC experts reached consensus that automatic referrals may improve the volume and timing of PC referrals, [14] with 86% of panelists suggesting that outpatient PC referrals should be based on both automatic referrals and clinician-based referrals [14]. There is limited research on the implementation of automatic referrals in practice. For instance, Adelson et al. [26] developed criteria for hospitalized patients with metastatic cancer that would trigger referral for a PC consultation as part of a pilot intervention. Their criteria were any one of advanced lung or pancreatic cancer diagnosis, prior hospitalization within 30 days, hospitalization for greater than 7 days, and the patients having active symptoms [26]. With their intervention, PC consultations doubled, and 30-day readmission rates decreased significantly [26]. However, the acceptability of automatic referrals among patients was not explored.

In a study by Zimmermann et al., [27] they assessed the feasibility of symptom screening in triggering early referral to PC. Patients completed the Edmonton Symptom Assessment System-revised (ESAS-r) to rate cancer symptoms such as pain, fatigue, drowsiness, nausea, anxiety, and depression from a scale of 0 (absent or best) to 10 (worst). Scores that were moderate to severe on more than one symptom triggered an email to the PC triage nurse who offered a PC clinic appointment to the patients. Zimmermann et al.’s [22] study revealed that patients who received early PC had improved mood and symptom control over time compared to those who did not receive early palliative care. However, Zimmermann et al. [22] also found in their study that a subset of patients who did not receive early PC still maintained good quality of life, mood, and symptom control. Therefore, the authors recommend that routine early PC may not be necessary for patients who report mild symptoms.

Nonetheless, prior to establishing thresholds for triggering consults, there is value in understanding whether “unselected” patients find an offer of consultation acceptable. In our co-design project, all team members identified that all patients newly diagnosed with advanced lung cancer might benefit from being offered a “Supportive and Palliative Care” consultation, regardless of the symptoms expressed and the prognosis and treatment options shared at the first visit. This was partly because the distress at diagnosis was perceived as widespread, and also because symptoms and disease trajectory can change quickly in lung cancer, necessitating early consultation so that patients are aware of how to activate further supports, such as home care, when needed later. Hence, early referral to supportive and palliative care regardless of prognosis and treatment options may help with identifying the varied needs of patients at the start of their cancer journey and lead to more sustainable care with less need for “crisis interventions” over time.

A strength of the co-design process was that it brought together people with disparate views about the meaning of PC. Our resulting process may avoid compounding patients’ potential fear of hearing or misunderstandings of the word “palliative” during their first oncology visit by instead being introduced to PC directly from the PC specialists who are comfortable framing and offering this added layer of support.

One of the limitations of our co-design study is that we have not captured the entire spectrum of clinicodemographic diversity among lung cancer patients and hence, despite the co-design process, the acceptability of an automatic call regarding palliative care may not be similarly accepted across patients of different ethnocultural and socioeconomic backgrounds, or patients undergoing different treatment types. Patients whose first language is not English, have different ethnocultural backgrounds, of different socio-economic backgrounds may have different perceptions of palliative care, and how acceptable an automatic referral process is. However, our co-design process is the first step to understanding the acceptability of an automatic referral for palliative care. This project will lead to the piloting of the program.

Following this pilot period, we will aim to survey and interview diverse lung cancer patients to understand the experiences and acceptability of this automatic referral process. In Paolucci et. al’s [19] systematic review of patient engagement in palliative care research, they only identified three studies that engaged patients with lived experience of cancer as partners in the health research process. In Abelson et. al’s [28] paper surveying patient partners across Canada, most of the patient partners who responded were white, female, born in Canada, and many were retired. For our project, we were able to recruit four patient advisors who identify as white, with lived experience with lung cancer and two family advisors who cared for patients with lived experience of cancer and palliative care. Engaging diverse patient partners who have varied ethnocultural and socioeconomic backgrounds has been the subject of focus of the patient partnership field, [29] and strategies to engage patient partners of diverse background remains a priority.

## Conclusion

Co-design ensures that stakeholders are involved in program development from the very beginning. It was especially valuable for this sensitive patient-provider topic, because it brought together various perspectives. Following the co-design process, we have implemented the automatic referral program in our cancer centre. The results of the evaluation of the acceptability and experience of early supportive and palliative care consultations from the perspectives of patients and caregivers will be reported elsewhere. Based on the success of this co-design process, we recommend involving patient advisors and clinicians in the design and evaluation of new health care services from the outset.

### Supplementary Information

**Supplementary Material 1.**

**Supplementary Material 2.**

**Supplementary Material 3.**

## Data Availability

Additional Data available on reasonable request.
