# Supplementary material for: Acceptability of automatic referrals to supportive and palliative care by patients living with advanced lung cancer: qualitative interviews and a co-design process
Source: Res Involv Engagem. 2024 Apr 2;10:36. doi: 10.1186/s40900-024-00568-0 (PMC10985851; doi:10.1186/s40900-024-00568-0)
Supplement: Supplementary file 1 — Supplementary Material 1. [file 40900_2024_568_MOESM1_ESM.docx]

**Appendix 1- Survey and Interview Guide developed with the co-design working group**

**Quantitative Survey**

Language line used? ❑ Yes ❑ No

Spoken to patient or family caregiver? ❑ Patient ❑ Family caregiver

Hello, can I talk to X? My name is ___. I’m from Alberta Health Services and I’m calling to ask your feedback on a recent call you had with [nurse name(s)] the supportive and palliative care nurse from Alberta Health Services.

Do you remember receiving a call from [nurse name] a supportive and palliative care nurse on ____?

(Another prompt: She may have offered to visit [you/your loved one] or sent you some information about supportive and palliative care?)

**Patient* doesn’t recall call:** Thank you for your time.

❑ Yes ❑ No

The supportive and palliative care phone call is a new way to contact patients, and we are eager to learn how acceptable it is. **Your feedback about the call will help to improve patient care and guide future planning of this program.** Participation in the survey is completely voluntary, confidential and will not affect your health care. It should take about 7 minutes to answer. As a thank you for participating, we would like to offer you a $20 gift card.* Would you like to participate and is now a good time for you?

*(gift card- can be anywhere that we can obtain digitally)

- Were [you/your loved one] expecting the call from the supportive care team?

❑ Yes ❑ No/not sure

- (*if yes to Q1*) How were [you/your loved one] told that [you/your loved one] would be getting the call? (check all that apply)

❑oncologist ❑oncology nurse ❑handout ❑not sure

**You’ll need to rate the next few questions from 1-5. I’ll read out the ratings after each question so you can choose your answer.**

| **Domain** | **Question** |
| --- | --- |
| **Assessment of Theoretical Framework of Acceptability domains** | |
| **Affective Attitude:** How individuals feel about the  phone call | 1. Overall, how acceptable was it to get a phone call from the supportive and palliative care nurse?   ❑ 1 = completely unacceptable ❑ 2 = somewhat unacceptable ❑ 3 = neither acceptable nor unacceptable  ❑ 4 = somewhat acceptable  ❑ 5 = completely acceptable  1.a (Ask in addition-**If talked to caregiver instead**) Overall, how acceptable do you think your loved one found the call?  ❑ 1 = completely unacceptable ❑ 2 = somewhat unacceptable ❑ 3 = neither acceptable nor unacceptable  ❑ 4 = somewhat acceptable  ❑ 5 = completely acceptable |
|  | thinking more about that phone call, I’m going to ask you how much do you agree or disagree with the following statements?  I’ll be using a 1-5 scale where 1 is completely disagree with the statement, and 5 is completely agree with the statement.   1. I felt comfortable getting a phone call from the supportive and palliative care nurse. (read out scale)   ❑ 1 = completely disagree  ❑ 2 = somewhat disagree  ❑ 3 = neither agree nor disagree  ❑ 4 = somewhat agree  ❑ 5 = completely agree |
| **Intervention coherence:** The extent to which the participant understands the phone call and how it works | I understood why I received a phone call offering [me/my loved one] a supportive and palliative care visit.  ❑ 1 = completely disagree  ❑ 2 = somewhat disagree  ❑ 3 = neither agree nor disagree  ❑ 4 = somewhat agree  ❑ 5 = completely agree |
| **Opportunity costs:** The extent to which benefits, values, must be given up to engage in the phone call | 1. It was a valuable use of my time to participate in the phone call.   ❑ 1 = completely disagree  ❑ 2 = somewhat disagree  ❑ 3 = neither agree nor disagree  ❑ 4 = somewhat agree  ❑ 5 = completely agree |
| **Perceived effectiveness:** The extent to which the phone call is likely to achieve its purpose | 1. I think talking to the supportive and palliative care nurse helped me.   ❑ 1 = completely disagree  ❑ 2 = somewhat disagree  ❑ 3 = neither agree nor disagree  ❑ 4 = somewhat agree  ❑ 5 = completely agree   1. I was able to learn about supportive and palliative care from the phone call.   ❑ 1 = completely disagree  ❑ 2 = somewhat disagree  ❑ 3 = neither agree nor disagree  ❑ 4 = somewhat agree  ❑ 5 = completely agree |
| **Ethicality:** The degree to which the phone call has good fit with an individual’s value system | 1. I was not concerned the supportive and palliative care nurse already had [my/my loved one’s] phone number and access to [my/my loved one’s] health information.   ❑ 1 = completely disagree  ❑ 2 = somewhat disagree  ❑ 3 = neither agree nor disagree  ❑ 4 = somewhat agree  ❑ 5 = completely agree |
| **Burden:** Perceived amount of effort required to participate in the (phone call) | 1. It didn’t take much emotional or physical effort to participate in the phone call.   ❑ 1 = completely disagree  ❑ 2 = somewhat disagree  ❑ 3 = neither agree nor disagree  ❑ 4 = somewhat agree  ❑ 5 = completely agree |
| **Self-efficacy:** The participants confidence that they can perform the behaviour(s) required to participate in the phone call | 1. During the phone call, you may have needed to listen, ask questions, and decide whether or not you wanted to meet with the supportive and palliative care nurse.   How much do you agree with the following statement:  I was confident in my abilities to participate in the phone call with the supportive and palliative care nurse.  ❑ 1 = completely disagree  ❑ 2 = somewhat disagree  ❑ 3 = neither agree nor disagree  ❑ 4 = somewhat agree  ❑ 5 = completely agree |
| **Overall acceptability assessment** | |
|  | 1. Did [you/your loved one] agree to the meet with the supportive and palliative care nurse?   ❑Yes ❑No ❑unsure: ____  **(***if yes to Q10***)** Have you already met the supportive and palliative care nurse?  ❑ Yes ❑ No |
| **Assessment of contextual and demographic factors** | |
| Questions on the meaning of palliative care | **I’m next going to ask you a few questions about palliative care.**   1. Before the phone call from the supportive care nurse, had you heard of palliative care?   ❑ Yes  ❑ No   1. Have you ever had a personal experience with palliative care? (read out options)   ❑ Yes, for myself  ❑ Yes, for a loved one (family) ________  ❑ No  (do not read): ‘Don’t know’  **How much do you agree or disagree with the following statements now?**   1. Palliative care means only end of life care   ❑ 1 = completely disagree  ❑ 2 = somewhat disagree  ❑ 3 = neither agree nor disagree  ❑ 4 = somewhat agree  ❑ 5 = completely agree  (do not read): ‘Don’t know’   1. Palliative care means an added layer of support anytime in an illness   ❑ 1 = completely disagree  ❑ 2 = somewhat disagree  ❑ 3 = neither agree nor disagree  ❑ 4 = somewhat agree  ❑ 5 = completely agree  (do not read): ‘Don’t know’   1. I think palliative care support is useful for me/my loved one now.   ❑ 1 = completely disagree ❑ 2 = somewhat disagree ❑ 3 = neither agree nor disagree  ❑ 4 = somewhat agree ❑ 5 = completely agree  (do not read): ‘Don’t know’  **Finally, I’m going to ask a few questions about you to make sure we’ve talked to a variety of people.** |
|  | 1. May I ask your age category? Please stop me when I reach your category.   ❑ 55 and under  ❑ 56-65  ❑ 66-75  ❑ 76 and older  ❑ Prefer not to answer   1. How do you identify in terms of Gender identity?: (more than one option can be said) **(don’t need to read out all)**   ❑ Woman   ❑ Man  ❑ Transgender Man  ❑ Transgender Woman   ❑ Non-Binary  ❑ Two-Spirited  ❑ I prefer another gender identity not listed above  ❑ Prefer not to answer  *[Write down if unsure*:____________]   1. Including yourself, how many people live in your household? ____   ❑Other:______ |
|  | 1. What’s the highest level of education you have received?  ❑ Some High School or less   ❑ High school graduate  ❑ Some college or college diploma/ trade school  ❑ Some university /University degree  ❑ Post Graduate degree   ❑ Prefer not to answer |
|  | 1. Which one of the following categories best describe your household income before taxes. **Please stop me when I reach your category or if you prefer not to answer.**   ❑ less than $30,000  ❑ $30,000 to just under $60,000  ❑ $60,000 to just under $100,000  ❑ $100,000 or more  ❑ Prefer not to answer |
|  | Besides English or French, do you speak any other language on a daily basis?  ❑ Yes, specify: ________________________  ❑ No  People in Canada come from many different cultural and racial backgrounds. How would you identify yourself?   \| ❑ White \| ❑ Indigenous (for example: First Nations, Metis etc) \| \| --- \| --- \| \| ❑ Chinese \| ❑ Black \| \| ❑ Filipino \| ❑ Latin American \| \| ❑ Arab \| ❑ West Asian (for example: Iranian, Afghan etc) \| \| ❑ Korean \| ❑ Japanese \| \| ❑ South Asian (for example: East Indian, Pakistani, Sri Lankan etc) \| \| \| ❑ Southeast Asian (for example: Vietnamese, Cambodian, Malaysian, Laotian etc) \| \| \| ❑ Other (please specify:____) \| \| |
|  | 1. What are the first three digits of your postal code?_________   ❑ Prefer not to answer  (*ask the following question if didn’t answer q6*)  We’re only asking your postal code to understand whether you live in an urban or rural area. Could you describe whether you live in….?  ❑ City (Calgary)  ❑ Towns around Calgary (metroinfluence) (e.g Airdrie,  Cochrane, Okotoks, Springbank, Chestermere Lake, Priddis)  ❑ Rural  **Other_______** |

**Patient Interview Guide**

| Questions | Potential Probes |
| --- | --- |
| 1. Would you be able to tell me a bit about your cancer, and what this diagnosis has been like for you? 2. Please tell me about your first contact with the supportive and palliative care nurse (you may have been contacted and visited at your home by <nurse name>). How were you contacted? What was that like?    1. How did you feel about receiving a phone call offering you a visit from the supportive and palliative care nurse?    2. How acceptable was it to offer [you] this visit over the phone? 3. How did you feel about the visit from the supportive and palliative care nurses? 4. What was the visit about? Was that what you were expecting a supportive and palliative care visit to be?      1. How did you feel about the visit being in your home instead of a cancer center? 2. What do you think about the timing of the visit? 3. What was your overall experience with the visit(s)? Were your concerns addressed during the visit?    1. What are your thoughts about the name of the service, “supportive and palliative care”?    2. What does palliative care mean to you?    3. Did that change as the visit went on? 4. Would you recommend supportive and palliative care for other patients? / How strongly would you recommend connecting with a supportive and palliative care nurse? 5. What supports are you using now? Who do you see? 6. Is there something else you’d like to share about the phone call, visit, the whole process, or anything we could do to improve the program? 7. Do you have any questions for me or would you like any additional information? | Do you recall getting a phone call from the nurse? How did that feel?  Did you receive more than one visit?  What resources and information were you given before and during the visit?  Would you have preferred a telephone or virtual visit?  What do you think about the timing of the visit in terms of where you were in your illness, shortly after your first visit to the cancer centre?  What would you suggest as a better time?  What did you think was helpful about the visit?  Was there anything you would change about the visit?  Was there anything that you did not like about the visit?  What occurred or support did you receive during that visit? |
