# Supplementary material for: Acceptability of automatic referrals to supportive and palliative care by patients living with advanced lung cancer: qualitative interviews and a co-design process
Source: Res Involv Engagem. 2024 Apr 2;10:36. doi: 10.1186/s40900-024-00568-0 (PMC10985851; doi:10.1186/s40900-024-00568-0)
Supplement: Supplementary file 2 — Supplementary Material 2. [file 40900_2024_568_MOESM2_ESM.docx]

**PaCES Automatic Process Diagram**

**Exclusion Criteria**

- Stage IV lung cancer NOT confirmed, or awaiting further results to confirm diagnosis

(even if confirmed at future visit, we will not be able to call the patient as they won’t be identified as a ‘new’ patient)

- Recurrence or progression as stage IV
- Already referred to/seen by/requested specialist palliative care (TBCC Palliative team, Palliative Home care, Community Palliative Consult Team).

EXCEPTION: If prior in-patient palliative consult has been completed but no outpatient palliative follow-up was planned.

**Inclusion Criteria:**

- Newly diagnosed advanced stage IV non-small cell lung cancer patients (Stage IV confirmed by oncologist or triage RN on their clinical documentation or verbally to PC provider), **AND**
- Have had their first oncology visit (where Stage IV confirmed prior to visit or during visit)
- Can be any of:
  - Disease therapy pending
  - Disease therapy confirmed
  - No disease modifying therapy
  - Patient declines future visits to cancer centre
- 18 years of age or older
- Able to communicate by phone (in any language)

**Patient accepts or declines support**

**Triage nurse identifies newly diagnosed patients in spreadsheet**

**Oncologist mentions in charting/consult note in ARIA Stage IV diagnosis and given patient handout**

**PC Provider screens for patient eligibility (see inclusion/exclusion)**

**PC Provider fills out PaCES record form 2 after consult & sends to researcher**

**If unable to visit patient at home, PC nurse books Phase 1 room at TBCC**

**Identification of Eligible Patients**

**Oncologist/Oncology nurse gives patient supportive care handout**

**Patient* asks to call back later**

**PC Provider makes note in ARIA (communication to oncology)**

**PC Provider sends patient summary of conversation, confirmation of consultation appointment, & resources**

**PC Provider gives patient* # xxx-xxx-xxxx to call in case of appt change**

**PC Provider obtains email/mailing address of patient***

**PC provider schedules consultation & lets them know to expect a research call**

**Patient* says yes to consult**

**PC Nurse Phone Call**

**Patient distress during phone call**

**PC Provider gives number to Access mental health: 403-943-1500**

**PC provider obtains email/mailing address of patient**

**PC Provider sends resource list to patient**

**PC Provider makes note in ARIA (communication to oncology)**

**PC Provider fills out PaCES record form 1**

**PC Nurse calls eligible patient* to offer consultation**

**PC Provider makes note in netcare/right-fax/regular fax (communication to palliative care, family doctor,homecare)**

**PC provider lets patient know to expect a research call**

**PC provider obtains email/mailing address of patient**

**PC Provider sends patient summary of conversation & resources**

**Patient* says no to consult**

**PC provider offers option of calling back at a later time/ gives patient phone number to call in case change of mind**

**PC Provider sends PaCES researcher name and phone number of patient contacted (after every patient or caregiver contact)**

**PC Nurse encourages use of AHS Language line for non-English speaking patients OR speaks with family caregiver**

**PC provider calls patient* again to offer consultation**

**Voicemail reached (after 3x attempt of patient contact)**

**PC provider leaves voicemail message for patient (includes 403-944-1616 to call back)**

**Patient* calls automated answering machine**

**Patient* leaves name, number, and preferred time to call back in answering machine**

**Unable to reach patient**

*patient and/or caregiver if patient unable to talk on the phone due to cognitive impairment

STOP

**PC Provider makes note in ARIA (communication to oncologist, palliative care team, homecare)**

**Patient doesn’t contact PC provider**

**(4 weeks no contact)**

STOP

**Research Evaluation**

**researcher sends patient* e-giftcard (via email or mail) (valued at $20)**

**Participant doesn’t consent to study**

**Researcher makes note of decline**

**researcher asks for verbal consent for participation in quantitative survey**

**Participant verbally consents to survey**

**researcher conducts survey about pc provider phone call**

**Researcher asks for verbal consent for qualitative interview (either same day or scheduled for later)**

**researcher sends completed consent form to patient* via email or mail**

**researcher provides information about the study, reviews consent form**

**Researcher phones patient* after pc provider call (1-2 days) afterwards)**

**researcher encourages use of AHS Language line for non-English speaking patients OR speaks with family caregiver**

STOP

**Researcher invites healthcare providers (pc nurse, oncologists/oncology nurse, social worker for qualitative interview (5-6 months after implementation)**

**researcher conducts interview**

**researcher obtains verbal consent**

**researcher reviews consent form with HCP**
